# Pre-sampling conditions affect salivary genomic DNA yield and human DNA fraction: a single-donor pilot study

**DOI:** 10.1186/s44342-026-00077-4

**Published:** 2026-07-20

**Authors:** Sangsoo Park, Hyoungjin Choi, Jong Bhak

**Affiliations:** 1 Korean Genomics Center, Ulsan National Institute of Science and Technology, Ulsan, 44919 Republic of Korea; 2 Department of Biomedical Engineering, College of Information-Bio Convergence Engineering, Ulsan National Institute of Science and Technology, Ulsan, 44919 Republic of Korea

**Keywords:** Genomic DNA, Saliva, Pre-sampling conditions, Human DNA fraction

## Abstract

As saliva-based genomic analyses expand, the effect of common pre-sampling behaviors on salivary genomic DNA (gDNA) recovery remains poorly characterized. In this single-donor pilot study, we tested eight pre-sampling conditions combining tooth brushing, gargling, and water intake. Saliva was collected in triplicate under each condition, cells were isolated, and gDNA was extracted using the Qiagen Kit. Three-way ANOVA showed that brushing was the dominant factor, accounting for 62.9% of total variation in DNA yield. Gargling had a smaller but significant effect (5.6%), while water intake had no significant effect. DNA purity, assessed by A260/280 ratio, remained stable across all conditions. To assess the human-to-bacterial DNA ratio, we performed qPCR using RPPH1 (human) and 16S rRNA (bacterial) markers. Brushing increased the human DNA fraction but reduced the absolute amount of human DNA recovered, indicating a purity-yield trade-off. These single-donor pilot findings offer preliminary, quantitative guidance for pre-sampling conditions in saliva-based genomic analyses, to be validated in multi-donor cohorts.

**Supplementary Information:**

The online version contains supplementary material available at 10.1186/s44342-026-00077-4.

## Introduction

Saliva is a non-invasive, easily accessible biospecimen that offers substantial promise for genomic and epigenomic applications [1]. Among its components, epithelial cells and leukocytes serve as a valuable source of gDNA, particularly for whole-genome sequencing, single-nucleotide polymorphism (SNP) detection, and structural variant analyses [2–6]. However, the quantity and quality of such gDNA, which are critical factors determining the efficiency of downstream genomics, can vary significantly depending on the donor’s pre-sampling behavior [7, 8].

Despite increasing adoption of saliva in clinical and research settings, systematic guidance on daily habits such as brushing, gargling, or water intake before collection is lacking [9]. Previous studies have examined individual pre-collection variables in isolation, but a systematic factorial comparison of multiple behaviors within a single experimental framework has not been reported. These behaviors can influence yield and purity. For instance, food or drink consumption dilutes salivary cell contents and alters protein composition, thereby reducing DNA recovery and compromising spectrophotometric purity measurements. Similarly, tooth brushing may alter the cellular and chemical composition of collected saliva, potentially interfering with extraction efficiency [7, 8, 10].

This study aims to characterize how pre-sampling behavior affects the yield, purity, and human-to-bacterial DNA composition of salivary gDNA by systematically comparing eight distinct pre-sampling conditions, each defined by a unique combination of brushing, gargling, and water intake. Through triplicate sampling and quantitative analysis, we assessed gDNA yield and A260/280 purity across conditions, and used qPCR with human (RPPH1) and bacterial (16S rRNA) markers to determine how these conditions affect the human-to-bacterial DNA ratio of the recovered gDNA. These results provide pilot, quantitative guidance for controlling pre-sampling variables in saliva gDNA workflows.

## Materials and methods

### Study design and pre-sampling conditions

All samples were collected from a healthy adult under eight pre-sampling conditions defined by three factors: tooth brushing (+/−), gargling (+/−), and water intake (+/−). Each condition was sampled in triplicate (*n* = 3). For the “brushing condition,” the donor used a standard toothbrush for 3 min. For the “mouthwash gargling condition,” the donor gargled with 20 mL of Listerine® Cool Mint (Johnson & Johnson, Skillman, NJ, USA) mouthwash for 30 s (product use guidelines) and expectorated without additional water rinsing. For the “water intake condition,” the donor drank 500 mL of water. Saliva was collected 1 h after completion of assigned procedures for all conditions to control for timing-related variability.

### Saliva collection and handling

Whole saliva, 5 mL, was collected in a 50-mL conical tube for each condition. All centrifugation steps were performed at room temperature (RT). Wide-bore pipette tips were used for resuspension and transfers to minimize mechanical shear.

### Isolation and washing of salivary cells

To isolate salivary cells, 6 volumes of 1 × phosphate-buffered saline (PBS) were added to 5 mL of saliva. The two were mixed gently by pipetting and rotating the tube at RT for 5 min. Samples were centrifuged at 500 × g for 5 min, and the supernatant was discarded. The pellet was resuspended in 5 mL 1 × PBS and washed again by repeating the mixing and centrifugation steps. The final pellet was resuspended in 1 mL 1 × PBS, and the suspension was aliquoted into four 1.5-mL microcentrifuge tubes. The tubes were centrifuged at 2000 × g for 10 min to pellet cells for gDNA extraction.

### Genomic DNA extraction

The Puregene® Blood Core Kit (Cat no. 158023; Qiagen, Hilden, Germany) was used to extract gDNA from saliva-derived cell pellets according to the manufacturer’s protocol.

#### Cell lysis

Pellets were lysed with 275 µL of Cell Lysis Solution and 1.5 µL of Proteinase K, mixed by inversion (25 times), and incubated at 55 ℃ for 1 h.

#### RNA removal

1.5 µL of RNase A Solution was added, mixed by inversion (25 times), and incubated at 37 ℃ for 15 min.

#### Protein precipitation

One hundred microliter of protein precipitation solution was added, vortexed for 20 s, incubated on ice for 5 min, and centrifuged at 16,000 × g for 3 min.

#### DNA precipitation

The supernatant was transferred to a new tube containing 300 µL of isopropanol, mixed by gentle inversion (50 times), incubated at RT (15–25 ℃) for 5 min, and centrifuged at 16,000 × g for 5 min.

#### DNA wash

The pellet was washed with 300 µL 70% ethanol, centrifuged at 16,000 × g for 1 min, and the ethanol was removed. The pellet was air-dried for ~ 5 min while avoiding over-drying.

#### DNA hydration

DNA was hydrated in 100 µL of DNA hydration solution, vortexed for 5 s at medium speed, and incubated at 65 ℃ for 1 h for dissolution.

### DNA quantification and purity assessment

DNA was quantified via Qubit fluorometry and a NanoDrop spectrophotometer. For fluorometric quantification, the Qubit dsDNA HS Assay Kit (Thermo Fisher Scientific, Waltham, MA, USA) was employed, and the manufacturer’s instructions were followed. Absorbance at 260 nm (A260) was used for spectrophotometric quantification, and purity was evaluated using the absorbance ratio at 260 and 280 nm (A260/A280). Total DNA yield (ng) was calculated based on the concentration and elution volume: 100 µL.

### Statistical analysis

All analyses were performed in triplicate per condition. Total DNA yield and A260/A280 values are summarized as mean ± standard deviation (SD). The coefficient of variation (CV, %) for total DNA yield under each condition was calculated as SD/mean × 100. To identify the pre-sampling factor that most strongly influences DNA yield, a three-way analysis of variance (ANOVA) was performed applying a 23 full-factorial design, brushing × gargling × water intake. Qubit fluorometry data were used as the primary outcome due to their specificity for double-stranded DNA. Effect sizes were reported as a percentage of total variation explained by each factor. All statistical data analyses and visualizations were performed using GraphPad Prism 11.0.0 (GraphPad Software, Boston, MA, USA). A *p*-value < 0.05 was considered statistically significant.

### qPCR-based quantification of human and bacterial DNA

To distinguish human from bacterial DNA in the extracted gDNA, qPCR was performed for each of the eight conditions. Human DNA was targeted with primers to the single-copy nuclear gene RPPH1 (forward: 5′-AGCTTGGAACAGACTCACGG-3′, reverse: 5′-AATGGGCGGAGGAGAGTAGT-3′), and bacterial DNA with universal 16S rRNA primers 1114 F (5′-CGGCAACGAGCGCAACCC-3′) and 1221R (5′-CCATTGTAGCACGTGTGTAGCC-3′). Reactions used Applied Biosystems SYBR Green master mix (Cat. No. A66732) with 400 nM primers and 10 ng total DNA per reaction. Because RPPH1 and 16S rRNA differ in genomic copy number, direct ΔCt comparison is biased; ΔCt (= Ct RPPH1 − Ct 16S) was converted to a human DNA proportion using an 11-point human-to-bacterial DNA ratio standard curve measured in triplicate (33 points) and fitted with a normalized logistic model (Top = 100, Bottom = 0; K = 0.5925, V₅₀ = 12.58; R2 = 0.9981; Fig. S2) [11]. Unknowns were interpolated in GraphPad Prism 11.0.0.

### Ethical approval

This study was approved by the Institutional Review Board of Ulsan National Institute of Science and Technology, Ulsan, South Korea (UNIST IRB; Approval No. UNISTIRB-15–19-A). All procedures were conducted per relevant guidelines. Written informed consent was pre-obtained from the participant.

## Results

### Brushing significantly reduces DNA yield

**Fig. 1:**
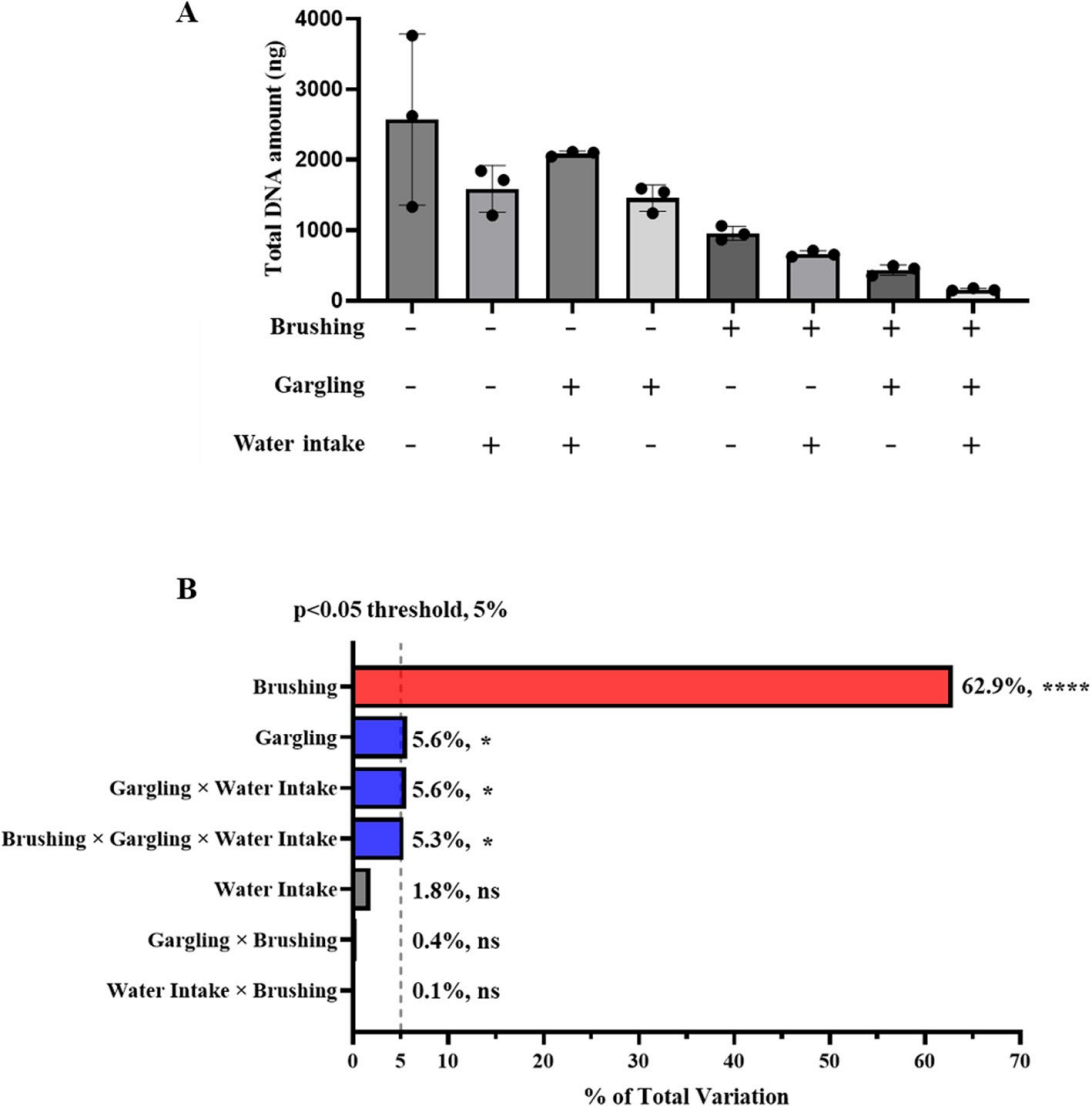
Effects of pre-collection behavior on saliva gDNA yield. **A** Total DNA (ng) across eight conditions combining brushing (+/−), gargling (+/−), and water intake (+/−). Bars show mean ± SD (*n* = 3). **B** Three-way ANOVA-based partitioning of variance in DNA yield. Brushing accounted for the largest proportion of total variation (62.9%, ****p-value < 0.0001), while gargling and selected interaction terms contributed smaller fractions (Gargling: 5.6%, *p-value = 0.0413, Water intake × Gargling: 5.6%, *p-value = 0.0427, Brushing × Gargling × Water intake, *p-value = 0.0466). Only water intake was insignificant (1.8%, ns). *p < 0.05; ns, not significant

Across the eight pre-collection conditions, DNA yield consistently differed by brushing status; the non-brushing condition produced higher yields, whereas the yield was reduced in the brushing-containing condition (Fig. 1A). To identify the main causative factor of yield differences, we used a three-way ANOVA to ascertain the proportion of the total yield variation which was explained by each factor and the interactions among them. Each percentage indicates the contribution of a given factor to the differences in DNA yield observed across the eight conditions. Brushing accounted for 62.9% of the total yield variation, meaning that changes in brushing status (brushing vs. no brushing) could explain 62.9% of the yield differences. Thus, brushing was the single most influential factor affecting DNA recovery under the tested pre-collection behaviors (Fig. 1B, Table. S1). The significant Water intake × Gargling interaction reflected a reversal in the direction of the gargling effect: without brushing, gargling reduced yield when no water was consumed (2570 to 1457 ng) but increased it when water was consumed (1587 to 2086 ng). This reversal was absent under brushing: gargling reduced yield regardless of water intake. This difference accounts for the significant three-way interaction. Reproducibility, assessed as the coefficient of variation across triplicates, varied among conditions but confirmed consistent recovery overall (Fig. S1, Table S2).

### Pre-sampling conditions do not affect DNA purity

**Fig. 2:**
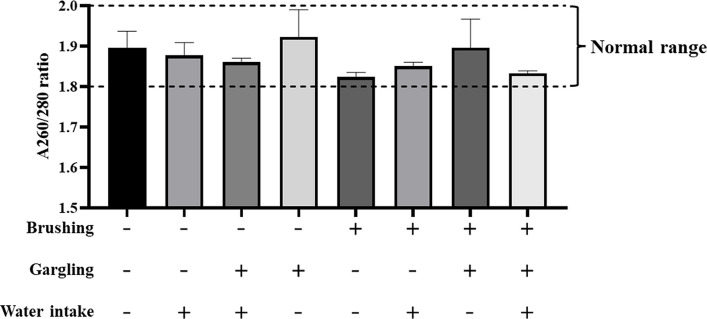
DNA purity across pre-sampling conditions was assessed using the A260/280 ratio. The A260/280 ratio of saliva-derived gDNA across pre-sampling conditions each +/−, eight conditions, was measured. Bars show the mean values of three replicate measurements, and error bars indicate the standard deviation (*n* = 3). Horizontal lines indicate the acceptable purity range of 1.8–2.0

DNA purity was evaluated based on the A260/280 ratio (Fig. 2). Across conditions, these ratios were within the acceptable range of 1.8–2.0, with no condition-specific deviations. These results indicate that pre-sampling interventions primarily affect the amount and variability of the recovered DNA, while measured DNA purity stabilizes across conditions.

### Brushing increases human DNA fraction but not human DNA recovery

**Fig. 3:**
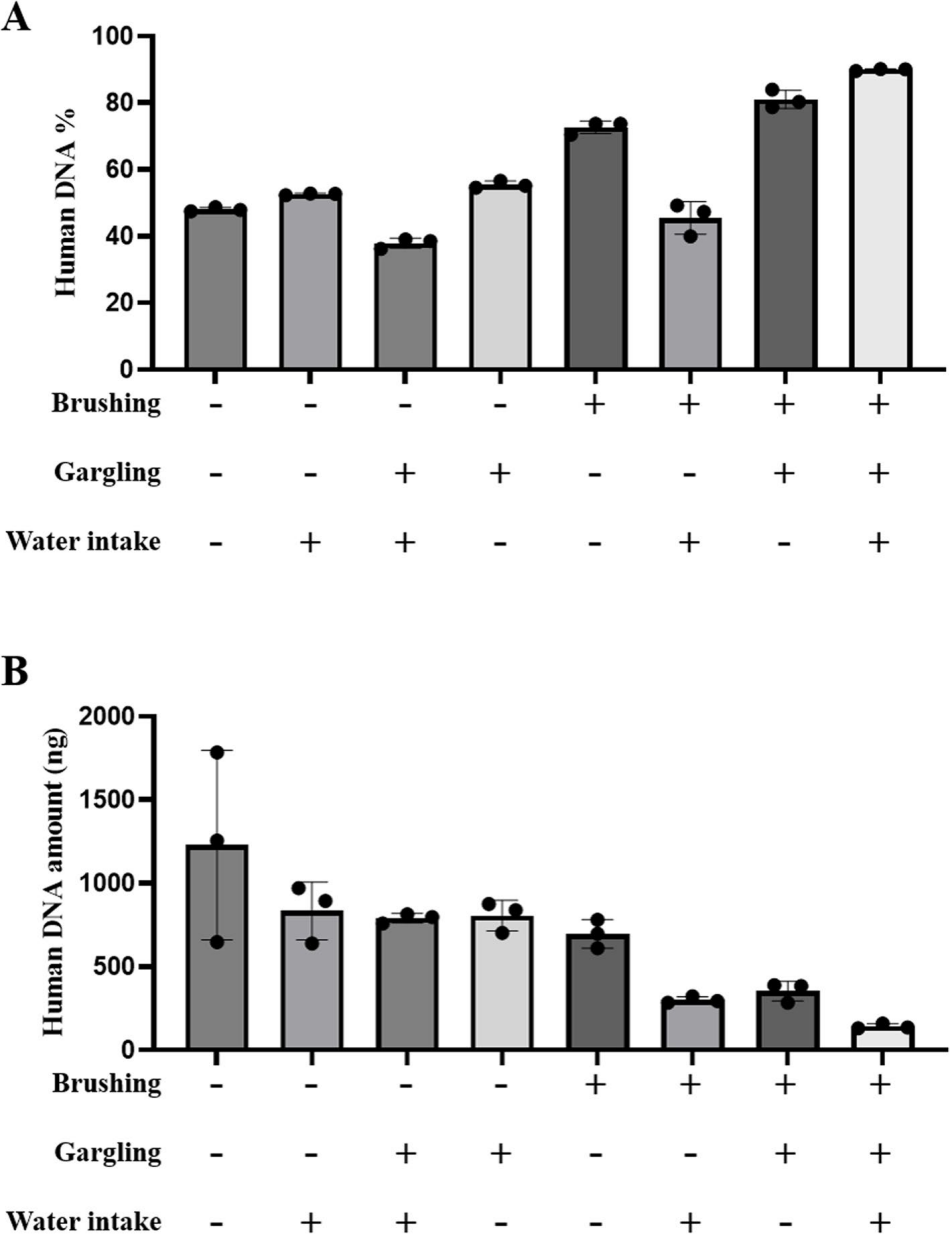
Human DNA fraction and absolute human gDNA amount across pre-sampling conditions. **A** Human DNA percentage of recovered gDNA, determined by qPCR (RPPH1 vs 16S rRNA) and interpolated from a human-to-bacterial DNA ratio standard curve (Fig. S2). **B** Absolute human gDNA amount (ng), calculated as total DNA yield × human DNA fraction. Bars show mean ± SD (*n* = 3); SD in (**B**) was propagated from yield and human fraction

Human (RPPH1) and bacterial (16S rRNA) DNA were quantified by qPCR, and ΔCt values were converted to human DNA proportions using a human-to-bacterial DNA ratio standard curve (R2 = 0.9981; Fig. S2). The human DNA fraction ranged from 37.9% to 89.9% across the eight conditions (Fig. 3A). Brushing-positive conditions showed higher human DNA fractions than brushing-negative conditions (mean 72.3% vs 48.5%), with the highest value in B + G + W + (89.9%). The absolute human gDNA amount, calculated as total DNA yield × human DNA fraction, ranged from 142 to 1234 ng (Fig. 3B). The highest and lowest human DNA fractions did not correspond to the highest and lowest absolute human gDNA amounts.

## Discussion

In this single-donor pilot, pre-sampling behavior affected salivary gDNA along three axes: total yield, human DNA fraction, and absolute human DNA recovery. Among the three factors tested, brushing was clearly the dominant one. It sharply reduced total gDNA yield (Fig. 1A), yet at the same time increased the human DNA fraction of what was recovered, from a mean of 48.5% under brushing-negative conditions to 72.3% under brushing-positive conditions, reaching 89.9% in B + G + W +. This pattern is consistent with the mechanical removal of bacterial biofilm and dental plaque, which would otherwise be co-extracted and counted together with human DNA in fluorometric quantification. It also directly addresses the concern that high-yield, no-brushing samples may simply carry a larger bacterial burden rather than more human DNA: the qPCR data show that the elevated crude yield of the no-brushing conditions is accompanied by a lower, not higher, human fraction. Because brushing lowered total yield while raising human purity, human DNA fraction and absolute human DNA recovery ranked in opposite directions across the eight conditions. The highest-purity condition (B + G + W +, 89.9%) yielded the least absolute human DNA (142 ng), whereas the no-intervention condition (B − G − W −, 48.0%) yielded the most (1234 ng).

The appropriate pre-sampling condition therefore depends on the intended downstream use, and the two measured outcomes point to different choices.

If the goal is to maximize the absolute amount of human gDNA, for example when input mass is limiting for library preparation or when multiple assays must be run from a single collection, brushing should be avoided. Non-brushing conditions recovered roughly two- to threefold more absolute human gDNA on average than brushing conditions (Fig. 3B). This is because the modest gain in human fraction conferred by brushing did not compensate for the substantial loss in total yield that accompanied it, so the net amount of recoverable human DNA was lower whenever brushing was applied.

If the goal is instead to maximize human DNA purity, for example, to reduce the proportion of microbial reads in whole-genome or exome sequencing and thereby improve the efficiency of human-genome coverage, brushing is preferable despite the yield cost. Brushing raised the human fraction to as high as 89.9% (B + G + W +), lowering the relative contribution of non-human DNA in the extracted material. In this case the trade-off is accepting a smaller absolute quantity of DNA in exchange for a cleaner, more human-enriched template.

The effect of gargling was not independent of the other factors. Gargling, which removes already-desquamated epithelial cells, reduced yield on its own, as observed under brushing. With prior water intake and no brushing, however, gargling increased yield, plausibly because hydration stimulates salivary flow and rehydrates the mucosa, allowing gargling to promote fresh desquamation rather than only washing cells away. Under brushing, mechanical abrasion had already dislodged much of the epithelial layer, abolishing this modulation. Each interaction term accounted for only ~ 5% of total variation, comparable to the main effect of gargling, and the present design cannot separate effects of this size from inter-day variation; this mechanism is therefore tentative.

Several limitations follow from the pilot design and should be addressed before these observations are generalized. First, all samples were derived from a single healthy donor. Inter-individual differences in oral epithelial turnover, salivary flow and viscosity, and baseline microbial load are expected to affect both DNA yield and human fraction [12], so the specific values and rankings reported here may not transfer to other individuals; effects that are strong in this donor could be weaker, absent, or even reversed in others. A validation cohort of multiple donors is therefore required before any condition can be recommended as a general standard. Second, the eight conditions were tested on separate days rather than in a balanced, randomized crossover design. As a result, the treatment effects are confounded with day-to-day biological variation such as diet, systemic hydration, gingival status, and circadian rhythm, and smaller effects in particular cannot be cleanly separated from this background variation. A randomized crossover design across multiple donors would be needed to confirm the effects observed here and to estimate their magnitude with confidence. Finally, the donor had no notable oral conditions such as gingivitis or xerostomia, which can alter epithelial shedding and salivary composition, so the applicability of these findings to such populations remains untested. Within these constraints, this study provides a preliminary but quantitative account of how brushing, gargling, and water intake jointly shape salivary gDNA yield, human purity, and human-to-bacterial composition in a single donor, and it establishes a qPCR-based workflow for human DNA quantification that can be applied directly to the larger, multi-donor studies needed to define broadly applicable sampling recommendations.

## Supplementary Information

Additional file 1: Fig. S1–S2 and Table S1–S5.

## Data Availability

All data generated and analyzed during this study are included in this published article and its supplementary information files. The complete raw datasets are provided as supplementary materials, comprising the three-way ANOVA summary for total gDNA yield (Table S1), the individual triplicate yield measurements and coefficients of variation for all eight pre-sampling conditions (Table S2), the qPCR standard curve raw data (Table S3), the per-condition qPCR cycle threshold values with the interpolated human DNA fractions (Table S4), and the absolute human gDNA recovery per condition (Table S5). The standard curve used for human DNA quantification is shown in Figure S2.

## Abbreviations

A260: Absorbance at 260 nm
A260/A280: Absorbance ratio at 260 and 280 nm
ANOVA: Analysis of variance
CV: Coefficient of variation
df: Degrees of freedom
F (dfₙ Dfₓ): F statistic with numerator (dfₙ) and denominator (dfₓ) degrees of freedom
gDNA: Genomic DNA
PBS: Phosphate-buffered saline
Rep: Replicate
RNase A: Ribonuclease A
RT: Room temperature
SD: Standard deviation
SNP: Single-nucleotide polymorphism
SS: Sum of squares

## References

[1] Song M, Bai H, Zhang P, Zhou X, Ying B. Promising applications of human-derived saliva biomarker testing in clinical diagnostics. Int J Oral Sci. 2023;15(1):2. 36596771 10.1038/s41368-022-00209-w PMC9810734

[2] Theda C, Hwang SH, Czajko A, Loke YJ, Leong P, Craig JM. Quantitation of the cellular content of saliva and buccal swab samples. Sci Rep. 2018;8(1):6944. 29720614 10.1038/s41598-018-25311-0 PMC5932057

[3] Dawes C. Estimates, from salivary analyses, of the turnover time of the oral mucosal epithelium in humans and the number of bacteria in an edentulous mouth. Arch Oral Biol. 2003;48(5):329–36. 12711376 10.1016/s0003-9969(03)00014-1

[4] Kvapilova K, Misenko P, Radvanszky J, Brzon O, Budis J, Gazdarica J, et al. Validated WGS and WES protocols proved saliva-derived gDNA as an equivalent to blood-derived gDNA for clinical and population genomic analyses. BMC Genomics. 2024;25(1):187. 38365587 10.1186/s12864-024-10080-0 PMC10873937

[5] Herzig AF, Velo-Suarez L, Le Folgoc G, Boland A, Blanche H, Olaso R, et al. Evaluation of saliva as a source of accurate whole-genome and microbiome sequencing data. Genet Epidemiol. 2021;45(5):537–48. 33998042 10.1002/gepi.22386

[6] Gudiseva HV, Hansen M, Gutierrez L, Collins DW, He J, Verkuil LD, et al. Saliva DNA quality and genotyping efficiency in a predominantly elderly population. BMC Med Genomics. 2016;9:17. 27052975 10.1186/s12920-016-0172-y PMC4823890

[7] Hughes SR, Chapleau RR. Comparing DNA quantity and quality using saliva collection following food and beverage consumption. BMC Res Notes. 2019;12(1):165. 30904022 10.1186/s13104-019-4211-6 PMC6431066

[8] Janovicova L, Holaniova D, Vlkova B, Celec P. Pre-analytical factors affecting extracellular DNA in saliva. Diagnostics (Basel). 2024;14(3):249. 10.3390/diagnostics14030249 PMC10855236 38337765

[9] Salfer B, Havo D, Kuppinger S, Wong DTW, Li F, Zhang L. Evaluating pre-analytical variables for saliva cell-free DNA liquid biopsy. Diagnostics (Basel). 2023;13(10):1665. 10.3390/diagnostics13101665 PMC10217087 37238150

[10] Mendoza AC, Volante BB, Hernandez ME, Mendoza CC, Pliego AF, Baptista Gonzalez HA, et al. Design of a protocol for obtaining genomic DNA from saliva using mouthwash: samples taken from patients with periodontal disease. J Oral Biol Craniofac Res. 2016;6(2):129–34. 27195211 10.1016/j.jobcr.2016.01.002 PMC4862237

[11] Marotz CA, Sanders JG, Zuniga C, Zaramela LS, Knight R, Zengler K. Improving saliva shotgun metagenomics by chemical host DNA depletion. Microbiome. 2018;6(1):42. 29482639 10.1186/s40168-018-0426-3 PMC5827986

[12] Baker JL, Mark Welch JL, Kauffman KM, McLean JS, He X. The oral microbiome: diversity, biogeography and human health. Nat Rev Microbiol. 2024;22(2):89–104. 37700024 10.1038/s41579-023-00963-6 PMC11084736

