# Supplementary material for: Pre-sampling conditions affect salivary genomic DNA yield and human DNA fraction: a single-donor pilot study
Source: Genomics Inform. 2026 Jul 20;24:15. doi: 10.1186/s44342-026-00077-4 (PMC13383544; doi:10.1186/s44342-026-00077-4)
Supplement: Supplementary file 1 — Additional file 1: Fig. S1–S2 and Table S1–S5. [file 44342_2026_77_MOESM1_ESM.docx]

**Supplementary materials**

**Figure S1. Reproducibility of salivary gDNA yield across pre-sampling conditions.**

Coefficient of variation (CV, %) of total gDNA yield for each of the eight pre-sampling conditions, calculated from triplicate measurements (n = 3). Conditions are defined by tooth brushing (B), gargling (G), and water intake (W) status (−, not performed; +, performed).


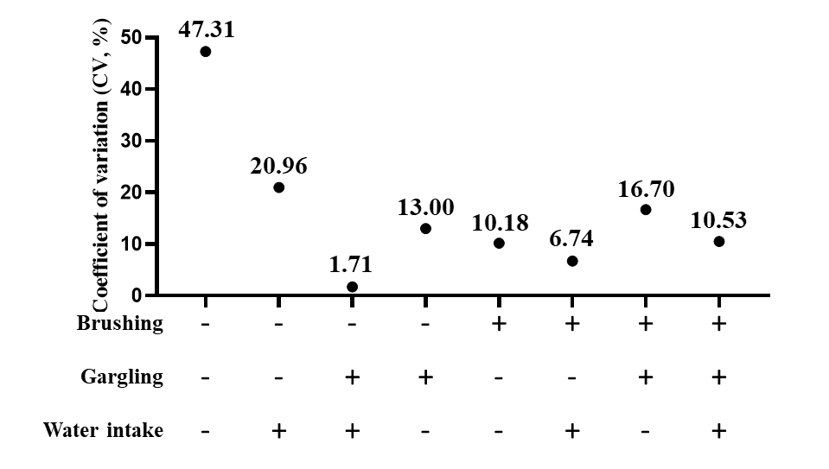


**Figure S2. Human Bacterial ratio DNA standard curve for qPCR-based human DNA quantification.**

Human DNA percentage as a function of ΔCt (Ct RPPH1 – Ct 16S) for 11 defined human-to-bacterial DNA ratio mixtures. Data were fitted with a two-parameter normalized logistic model (Top = 100, Bottom = 0): $Human DNA \left( \% \right)=\frac{100}{1+e^{(\Delta Ct-V50)}}$ with K = 0.5925 (95% CI 0.5729-0.6129), V50 = 12.58 (95% CI 12.53-12.63), and R² = 0.9981. Human DNA proportions for all experimental conditions were interpolated from this curve.

Abbreviations: CI, Confidence Interval

**Table S1.** Three-way ANOVA summary for salivary total DNA yield (Qubit). Statistical analysis was performed using a 2³ full-factorial design (Brushing × Gargling × Water Intake) with Type III sums of squares (n = 3 per condition, df*residual* = 16). ****p < 0.0001; *p < 0.05; ns, not significant.

| Source of Variation | SS | df | F  (dfₙ, dfₓ) | p-value | % of Total Variation |
| --- | --- | --- | --- | --- | --- |
| Brushing | 11,281,959 | 1 | F (1, 16) = 54.94 | < 0.0001 **** | 62.9% |
| Gargling | 1,010,651 | 1 | F (1, 16) = 4.92 | 0.041 * | 5.6% |
| Water Intake | 321,322 | 1 | F (1, 16) = 1.57 | 0.229 ns | 1.8% |
| Water Intake × Gargling | 995,115 | 1 | F (1, 16) = 4.85 | 0.043 * | 5.6% |
| Water Intake × Brushing | 17,658 | 1 | F (1, 16) = 0.086 | 0.773 ns | 0.1% |
| Gargling × Brushing | 63,963 | 1 | F (1, 16) = 0.311 | 0.585 ns | 0.4% |
| Water × Gargling × Brushing | 954,807 | 1 | F (1, 16) = 4.65 | 0.047 * | 5.3% |
| Residual | 3,285,824 | 16 |  |  |  |

Abbreviations: SS, sum of squares; df, degrees of freedom; F (dfₙ, dfₓ), F-statistic with numerator (dfₙ) and denominator (dfₓ) degrees of freedom*.*

**Table S2.** Coefficient of variation (CV%) of salivary total DNA yield (Qubit) across all pre-sampling conditions. Data represent individual replicate values and mean ± CV% from triplicate measurements (n = 3 per condition). Conditions are defined by tooth brushing, gargling, and water intake status (−, not performed; +, performed).

| Brushing | Gargling | Water Intake | Rep 1 (ng) | Rep 2 (ng) | Rep 3 (ng) | Mean (ng) | CV (%) |
| --- | --- | --- | --- | --- | --- | --- | --- |
| − | − | − | 2,620 | 1,330 | 3,760 | 2,570 | 47.31 |
| − | − | + | 1,710 | 1,840 | 1,210 | 1,587 | 20.96 |
| − | + | − | 1,240 | 1,540 | 1,590 | 1,457 | 12.99 |
| − | + | + | 2,100 | 2,045 | 2,112 | 2,086 | 1.71 |
| + | − | − | 866 | 1,060 | 946 | 957 | 10.18 |
| + | − | + | 624 | 654 | 712 | 663 | 6.74 |
| + | + | − | 494 | 458 | 354 | 435 | 16.70 |
| + | + | + | 177 | 146 | 151 | 158 | 10.53 |

Abbreviations: Rep, replicate; CV, coefficient of variation.

**Table S3.** qPCR standard curve raw data for human DNA quantification. Cycle threshold (Ct) values for the human single-copy gene (RPPH1) and the universal bacterial 16S rRNA gene, and the derived ΔCt (Ct RPPH1 – Ct 16S), for 11 defined human-to-bacterial DNA ratio gDNA mixtures (n = 3 per standard). ΔCt values were fitted to the two-parameter sigmoidal standard curve shown in Figure S2.

| Standard  (Human:Bacteria) | RPPH1 Ct (mean) | 16S Ct (mean) | ΔCt (mean ± SD) |
| --- | --- | --- | --- |
| Standard 1  (100:0) | 26.87 | 28.53 | -1.66 ± 0.06 |
| Standard 2  (90:10) | 27.12 | 18.42 | 8.69 ± 0.06 |
| Standard 3  (80:20) | 27.21 | 17.04 | 10.17 ± 0.02 |
| Standard 4  (70:30) | 27.23 | 15.94 | 11.29 ± 0.02 |
| Standard 5  (60:40) | 27.47 | 15.56 | 11.91 ± 0.10 |
| Standard 6  (50:50) | 27.66 | 15.13 | 12.52 ± 0.06 |
| Standard 7  (40:60) | 28.28 | 14.91 | 13.37 ± 0.12 |
| Standard 8  (30:70) | 28.69 | 14.79 | 13.90 ± 0.20 |
| Standard 9  (20:80) | 29.31 | 14.49 | 14.82 ± 0.03 |
| Standard 10  (10:90) | 30.85 | 14.31 | 16.54 ± 0.01 |
| Standard 11  (0:100) | 35.03 | 14.15 | 20.88 ± 0.08 |

Abbreviations: Ct, cycle threshold; ΔCt, delta Ct; SD, standard deviation.

**Table S4.** Per-condition qPCR data and interpolated human DNA fraction. Mean Ct values for RPPH1 and 16S rRNA, ΔCt (Ct RPPH1 – Ct 16S), and human DNA percentage interpolated from the standard curve (Figure S2) for each pre-sampling condition (n = 3 per condition). Conditions are defined by tooth brushing (B), gargling (G), and water intake (W) status (−, not performed; +, performed).

| Condition  (B, G, W) | RPPH1 Ct (mean) | 16S Ct (mean) | ΔCt (mean ± SD) | Human DNA % (mean ± SD) |
| --- | --- | --- | --- | --- |
| B− G− W− | 25.02 | 12.30 | 12.71 ± 0.04 | 48.0 ± 0.6 |
| B− G− W+ | 24.96 | 12.55 | 12.41 ± 0.02 | 52.6 ± 0.3 |
| B− G+ W− | 25.04 | 12.83 | 12.21 ± 0.07 | 55.4 ± 1.1 |
| B− G+ W+ | 25.66 | 12.25 | 13.41 ± 0.11 | 37.9 ± 1.5 |
| B+ G− W− | 23.81 | 12.88 | 10.93 ± 0.16 | 72.6 ± 1.9 |
| B+ G− W+ | 25.39 | 12.50 | 12.89 ± 0.34 | 45.5 ± 4.9 |
| B+ G+ W− | 24.58 | 14.46 | 10.12 ± 0.30 | 81.0 ± 2.7 |
| B+ G+ W+ | 24.05 | 15.16 | 8.89 ± 0.06 | 89.9 ± 0.3 |

Abbreviations: Ct, cycle threshold; ΔCt, delta Ct; SD, standard deviation; B, brushing; G, gargling; W, water intake.

**Table S5.** Absolute human gDNA recovery per pre-sampling condition. Human gDNA amount (ng) was calculated as total gDNA yield (Qubit) multiplied by the interpolated human DNA fraction. Errors on human gDNA (ng) were obtained by propagating the relative standard deviations of total yield and human DNA fraction (n = 3 per condition).

| Condition  (B, G, W) | Total gDNA yield  (ng, mean ± SD) | Human DNA (%, mean) | Human gDNA  (ng, mean ± SD) |
| --- | --- | --- | --- |
| B− G− W− | 2,570 ± 1,216 | 48.0 | 1,234 ± 584 |
| B− G− W+ | 1,587 ± 333 | 52.6 | 834 ± 175 |
| B− G+ W− | 1,457 ± 189 | 55.4 | 807 ± 106 |
| B− G+ W+ | 2,086 ± 36 | 37.9 | 790 ± 35 |
| B+ G− W− | 957 ± 97 | 72.6 | 695 ± 73 |
| B+ G− W+ | 663 ± 45 | 45.5 | 301 ± 38 |
| B+ G+ W− | 435 ± 73 | 81.0 | 352 ± 60 |
| B+ G+ W+ | 158 ± 17 | 89.9 | 142 ± 15 |

Abbreviations: SD, standard deviation; B, brushing; G, gargling; W, water intake.
